# Supplementary material for: Protrudin functions from the endoplasmic reticulum to support axon regeneration in the adult CNS
Source: Nat Commun. 2020 Nov 5;11:5614. doi: 10.1038/s41467-020-19436-y (PMC7645621; doi:10.1038/s41467-020-19436-y)
Supplement: Supplementary file 1 — Supplementary Information [file 41467_2020_19436_MOESM1_ESM.pdf]

Supplementary Information

Supplementary Figures

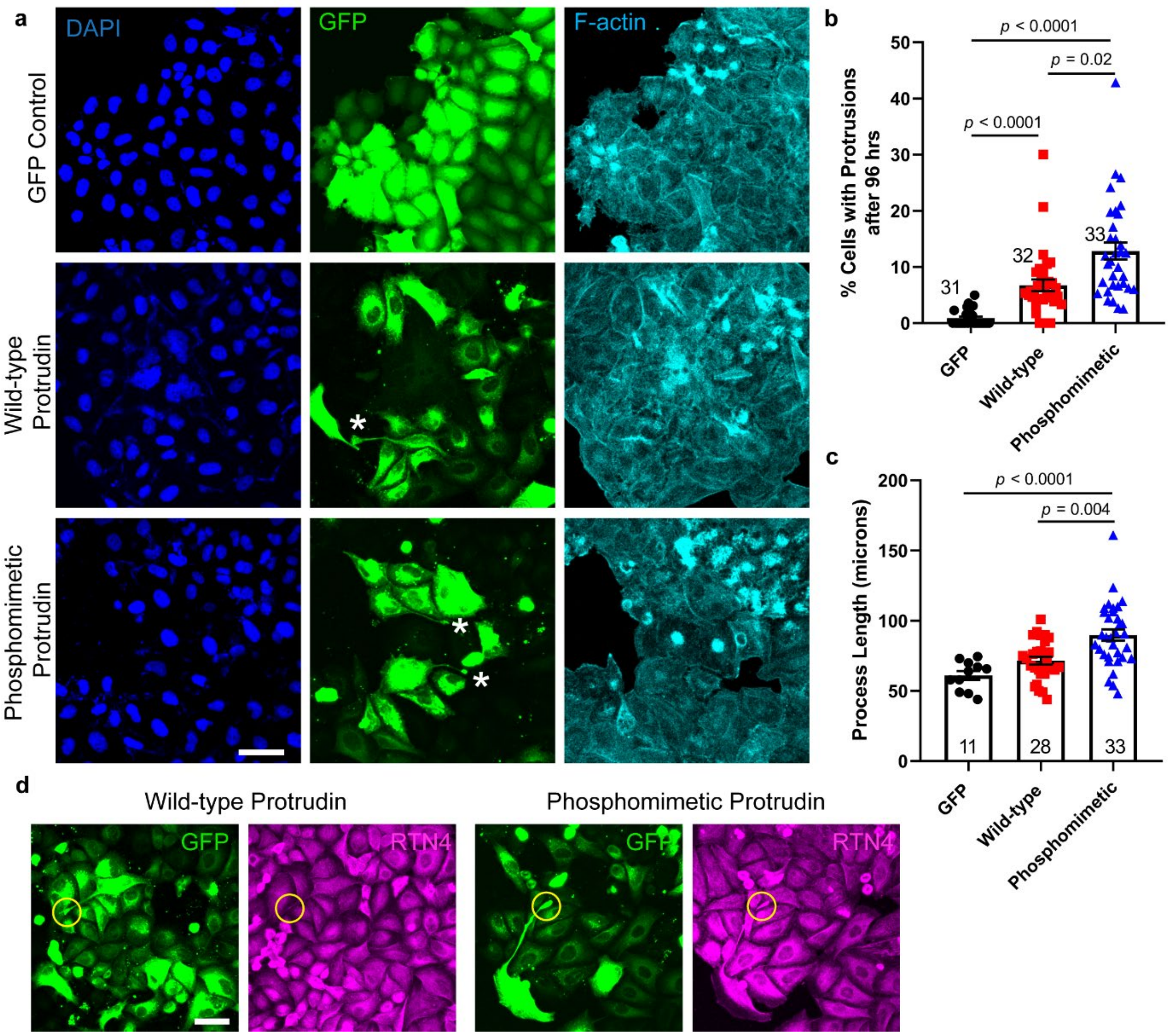

Supplementary Figure 1. Phosphomimetic Protrudin enhances HeLa cell protrusion formation. (**a**) Immunofluorescent images representing HeLa cells fixed 96 hours post transfection with GFP constructs (green) and stained for DAPI (blue) and F-actin (cyan). Scale bar is 50 μm. White asterisks represent the tip of protrusions. (**b**) The % of transfected cells which formed protrusions is higher in cells overexpressing phosphomimetic Protrudin compared to control or wild-type Protrudin (n = 3 independent experiments, $p < 0.0001$, *Kruskal-Wallis with Dunn's* multiple comparison test, *Kruskal-Wallis* statistic = 57.38). n on

the graph represents the number of images analysed per condition. Error bars represent mean ± SEM. (**c**) The length of the protrusions in cells overexpressing phosphomimetic Protrudin is greater than that of cells overexpressing control or wild-type Protrudin (n = 3 independent experiments, $p < 0.0001$, *Kruskal-Wallis with Dunn's* multiple comparison test, *Kruskal-Wallis* statistic = 20.95). n on the graph represents the number of cells analysed per condition. Error bars represent mean ± SEM. (**d**) Representative images to show an observed enrichment of RTN4 at the tip of protrusions in cells overexpressing phosphomimetic Protrudin but not wild-type Protrudin. Scale bars are 50 μm.

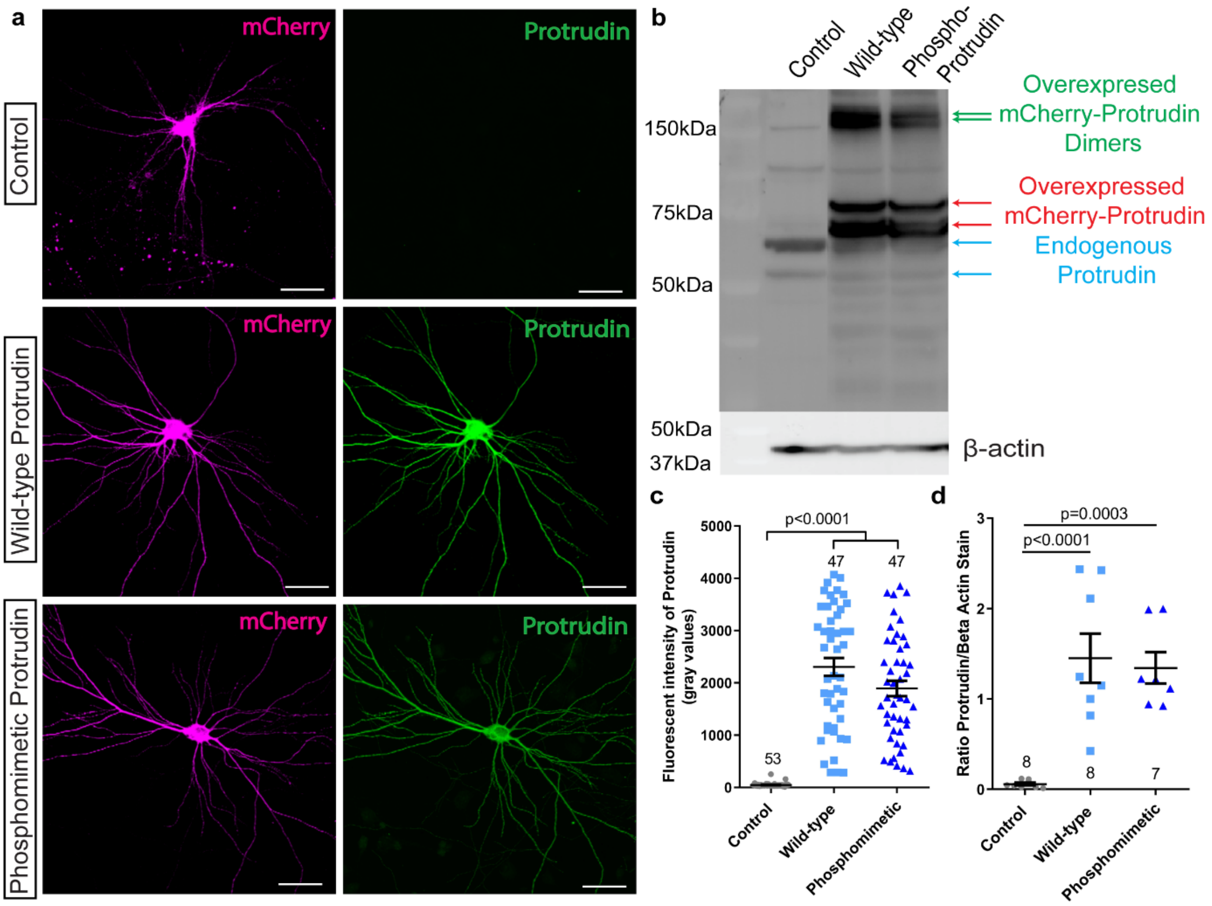

Supplementary Figure 2. Overexpression of Protrudin results in higher amount of the Protrudin protein in rat cortical neurons at 14 DIV. (**a**) Images showing mCherry fluorescence (magenta) and Protrudin immunofluorescence (green) from neurons transfected with control, wild-type or phosphomimetic Protrudin (magenta) and labelled with anti-Protrudin antibody. Scale bars are 20 µm. (**b**) Western blot showing Protrudin expression levels in PC12 cells overexpressing the three different constructs. The blot was stripped and probed for beta actin (lower panels). Blue arrows indicate endogenous Protrudin, red arrows indicate overexpressed Protrudin-mCherry and green arrows show overexpressed mCherry-tagged Protrudin dimers. (**c**) Scatter plot to show the average fluorescence intensity of Protrudin in transfected neurons (n = 3 independent experiments, n on graph represents the number of cells analysed per condition, *Kruskal-Wallis with Dunn's* multiple comparison test, $p < 0.0001$, *Kruskal-Wallis* statistic = 102.3). Error bars represent mean ± SEM. (**d**) Scatter plot to show the ratio of

Protrudin to beta actin staining of PC12 lysate extracted from cells overexpressing either control, wild-type or phosphomimetic Protrudin (n = 7-8 samples, *one-way ANOVA with Tukey's* multiple comparison test, $p < 0.0001$, F statistic = 17.44). Error bars represent mean ± SEM.

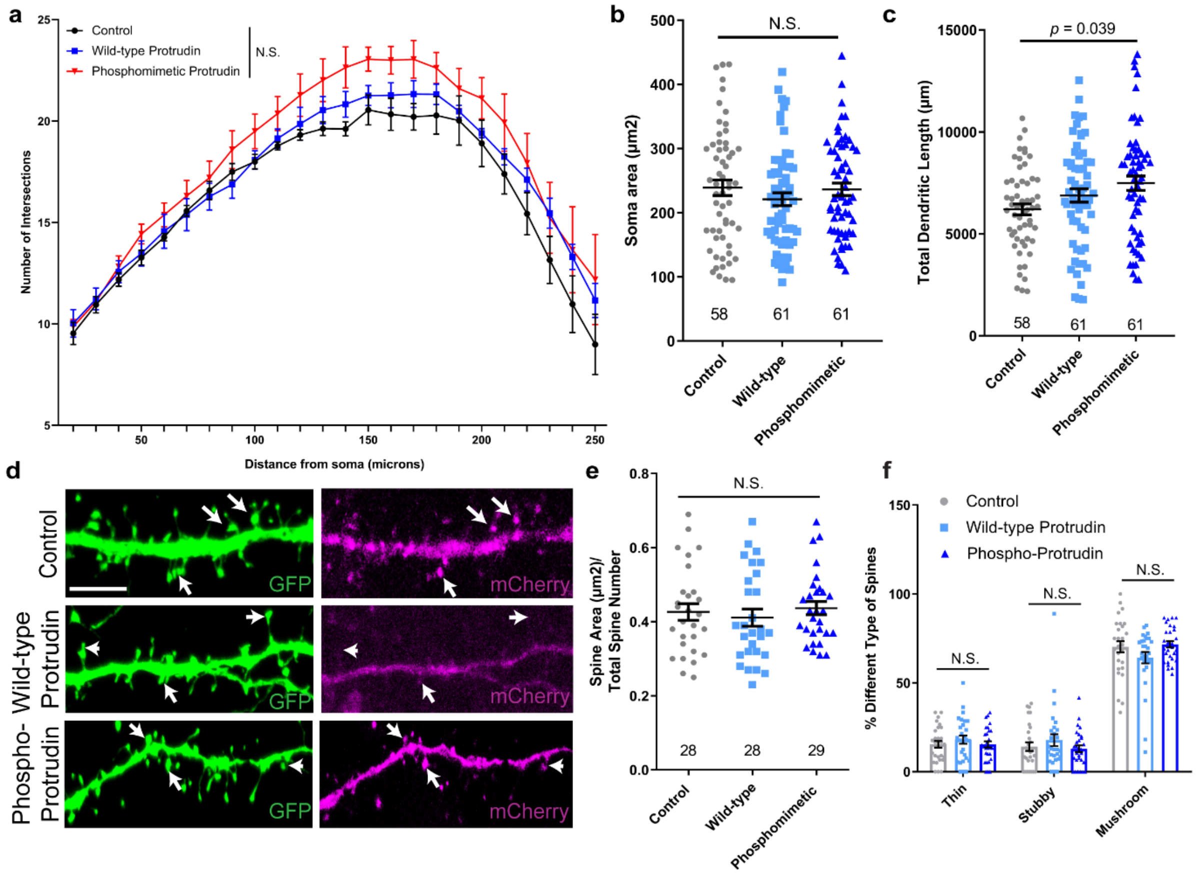

Supplementary Figure 3. Overexpression of Protrudin does not result in morphological changes. (**a**) Dendritic tree complexity of neurons transfected with each construct - the number of intersections at each distance from the cell soma was plotted for each condition. There are no significant differences between the conditions (n = 4 independent experiments, repeated measures *two-way ANOVA with Tukey's* multiple comparison test). Error bars represent mean ± SEM. (**b**) Average soma area in cells expressing the three different mCherry constructs (n = 5 independent experiments, n on graph represents number of cells analysed) (*Kruskal-Wallis with Dunn's* multiple comparison test, p = 0.142, *Kruskal-Wallis* statistic = 5.444). Error bars represent mean ± SEM. (**c**) Dendritic tree total length across the different conditions. Cells overexpressing phosphomimetic Protrudin have a more complex dendritic structure than cells overexpressing control (n = 4 independent experiments, n on graph represents the number of cells analysed) (*one-way ANOVA with Tukey's* multiple comparison test, p = 0.068, F-statistic = 2.409). Error bars represent mean ± SEM. (**d**) Representative z-project images of 20 µm z-

stack sections examined for dendritic spine number and morphology. White arrows point to individual spines. Scale bars are 5 μm. (**e**) The spine area (μm^2) per the total number of spines for each condition. There were no significant differences between the three conditions (n = 2 independent experiments, p = 0.509, *Kruskal-Wallis with Dunn's* multiple comparison test, *Kruskal-Wallis* statistic = 1.349). Error bars represent mean ± SEM. (**f**) There are no changes in spine morphology between the different conditions (n = 28 cells for control, n = 26 cells for wild-type and n = 29 cells for phosphomimetic Protrudin in 2 independent experiments) (*one-way ANOVA with Tukey's* multiple comparison test, p = 0.66 for thin, p = 0.51 for stubby and p = 0.17 for mushroom spines; F statistic = 0.541, 0.780, 1.689 respectively). Error bars represent mean ± SEM.

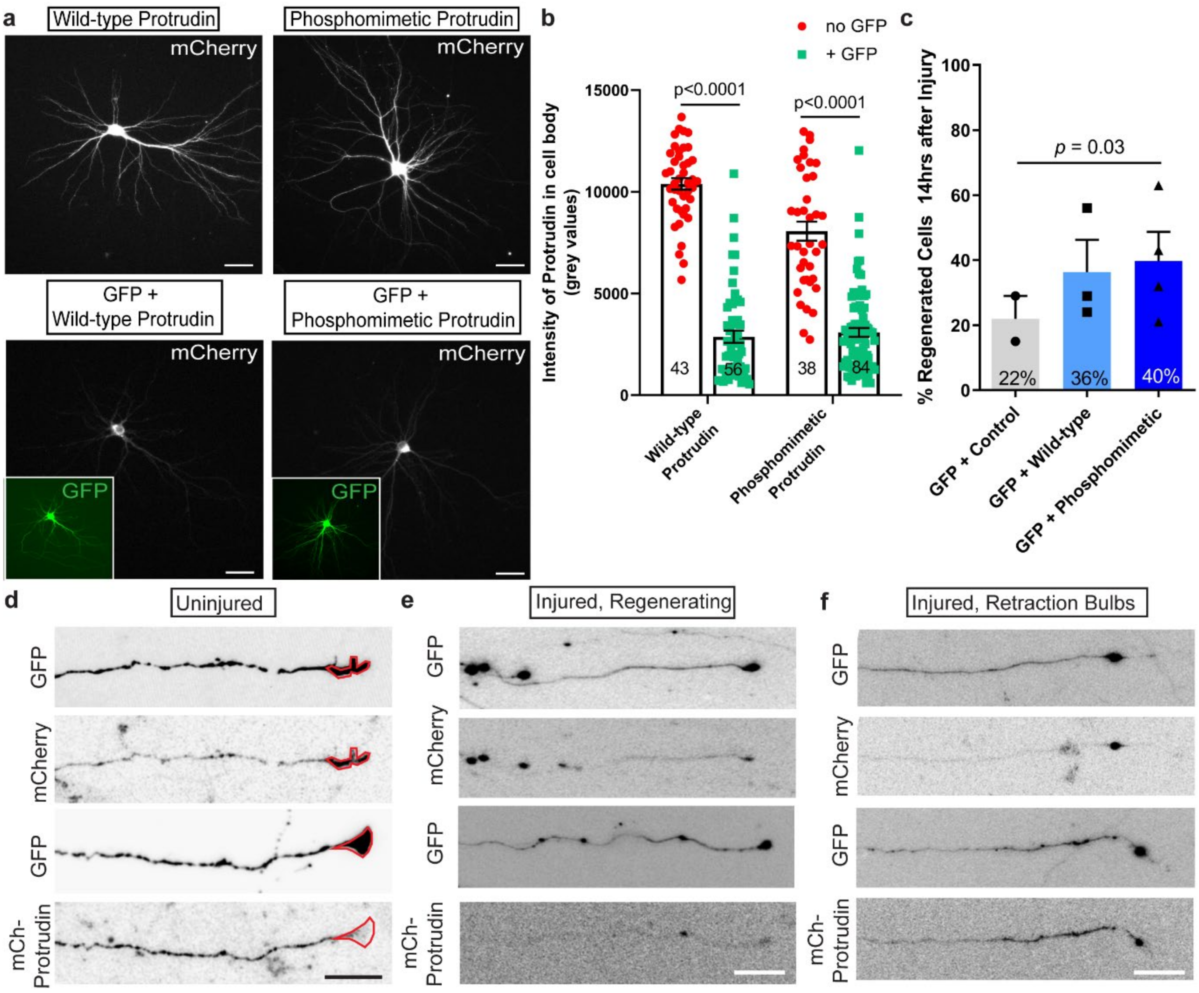

Supplementary Figure 4. Protrudin promotes regeneration in a dose-dependent manner and localizes to the axon tip. (**a**) Immunofluorescence images of cells expressing wild-type or phosphomimetic Protrudin fused to mCherry (white) on their own or in combination with GFP (green) control plasmid. Scale bars are 20 µm. (**b**) Quantification of mCherry-Protrudin fluorescence intensity in the soma. (*n* = 3 independent experiments, *n* on graph represent the number of cells analysed, Two-tailed *Student's* t-test). Error bars represent mean ± SEM. (**c**) Percentage of regenerating axons after laser axotomy in neurons co-transfected with Protrudin constructs and GFP (*n* = 2 independent experiments for control, *n* = 3 independent experiments for wild-type and *n* = 4 independent experiments for phosphomimetic Protrudin, at least 47 cells analyzed in each condition) (*Fisher's exact* test with analysis of stack of p values and Bonferroni-Dunn multiple comparison test). Error bars represent mean ± SEM. (**d**)

Representative images showing GFP and mCherry-Protrudin at the growth cone of neurons co-expressing both constructs. Red lines indicate the area of the growth cone as defined by the GFP signal. Scale bars are 10 µm. (**e**) Images showing regenerating axons after injury. Scale bars are 20 µm. (**f**) Images showing retraction bulbs of non-regenerating processes after laser axotomy. Scale bars are 20 µm.

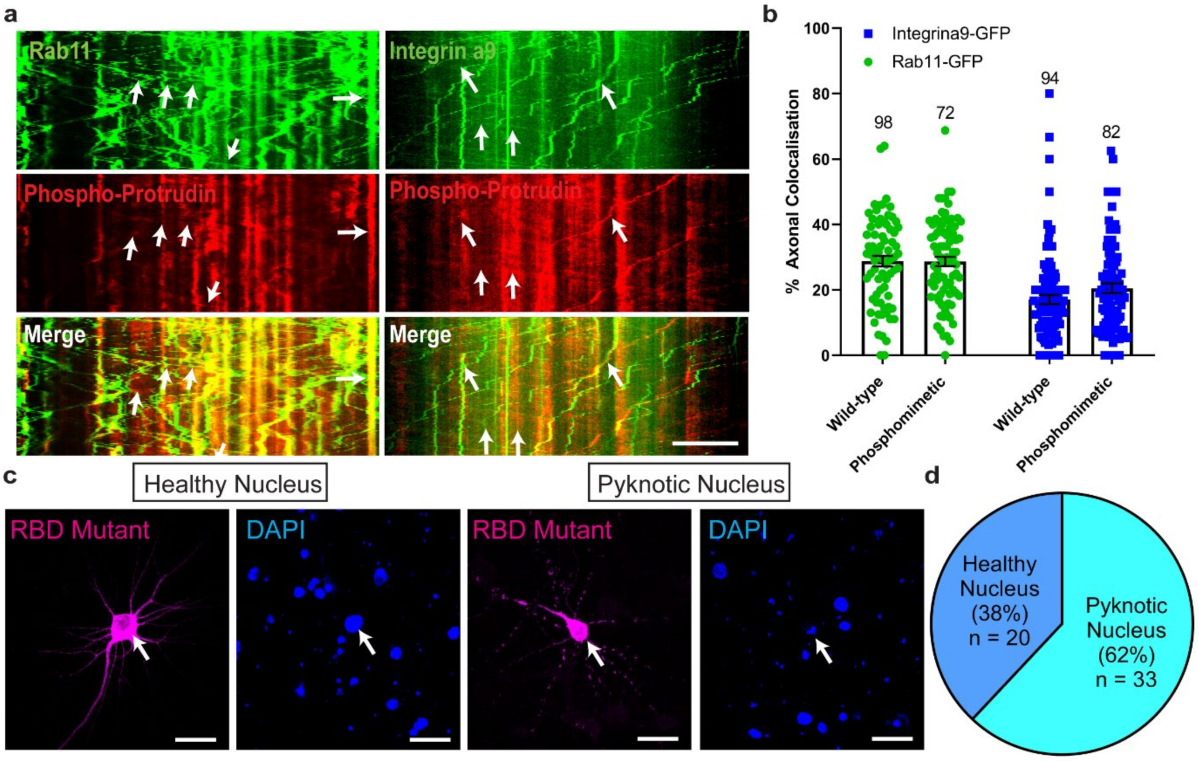

Supplementary Figure 5. Rab11 and integrin vesicles co-localize with Protrudin and RBD domain deletion has consequences on cell survival. (**a**) Kymographs showing co-localization between Rab11 or integrin α9-GFP with Protrudin. White arrows show areas of colocalization. Scale bar is 5 µm. (**b**) Wild-type and phosphomimetic Protrudin co-localize to similar extent with either Rab11 or integrin alpha 9. Error bars represent mean ± SEM. (**c**) Representative fluorescent images of RBD mutant of Protrudin in surviving and non-surviving (the most common phenotype) cells as shown by nuclear fragmentation (pyknosis). Scale bars are 20 µm. (**d**) Pie chart showing the percentage of cells with healthy and pyknotic nuclei in cortical neurons overexpressing RBD mutant protrudin (*n* = 3 independent experiments, *n* on the pie chart represent the number of cells analysed).

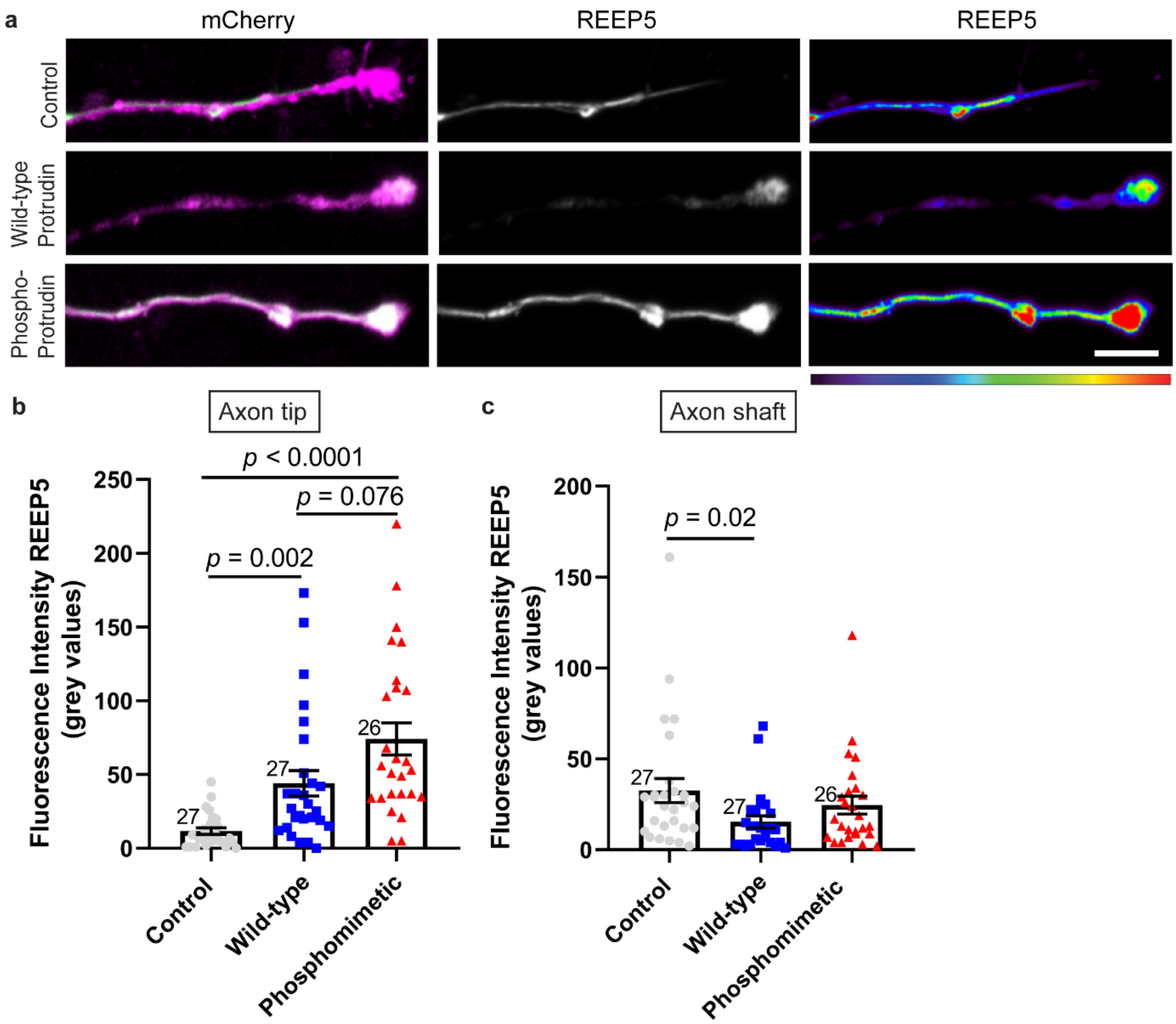

Supplementary Figure 6. Protrudin overexpression enhances ER presence at growth cones. (**a**) Representative images of REEP5-GFP fluorescence (grey) in the distal axon of neurons expressing the indicated m-Cherry control and Protrudin constructs (magenta). Scale bar is 10 µm. (**b**) Quantification of REEP5 fluorescence intensity at the axon tip ($p < 0.0001$, *Kruskal-Wallis with Dunn's* multiple comparisons test, *Kruskal-Wallis* statistic = 32.80). Error bars represent mean ± SEM. (**c**) Quantification of REEP5 fluorescence intensity at the axon shaft ($p = 0.02$, *Kruskal-Wallis with Dunn's* multiple comparisons test, *Kruskal-Wallis* statistic = 7.873). Error bars represent mean ± SEM.

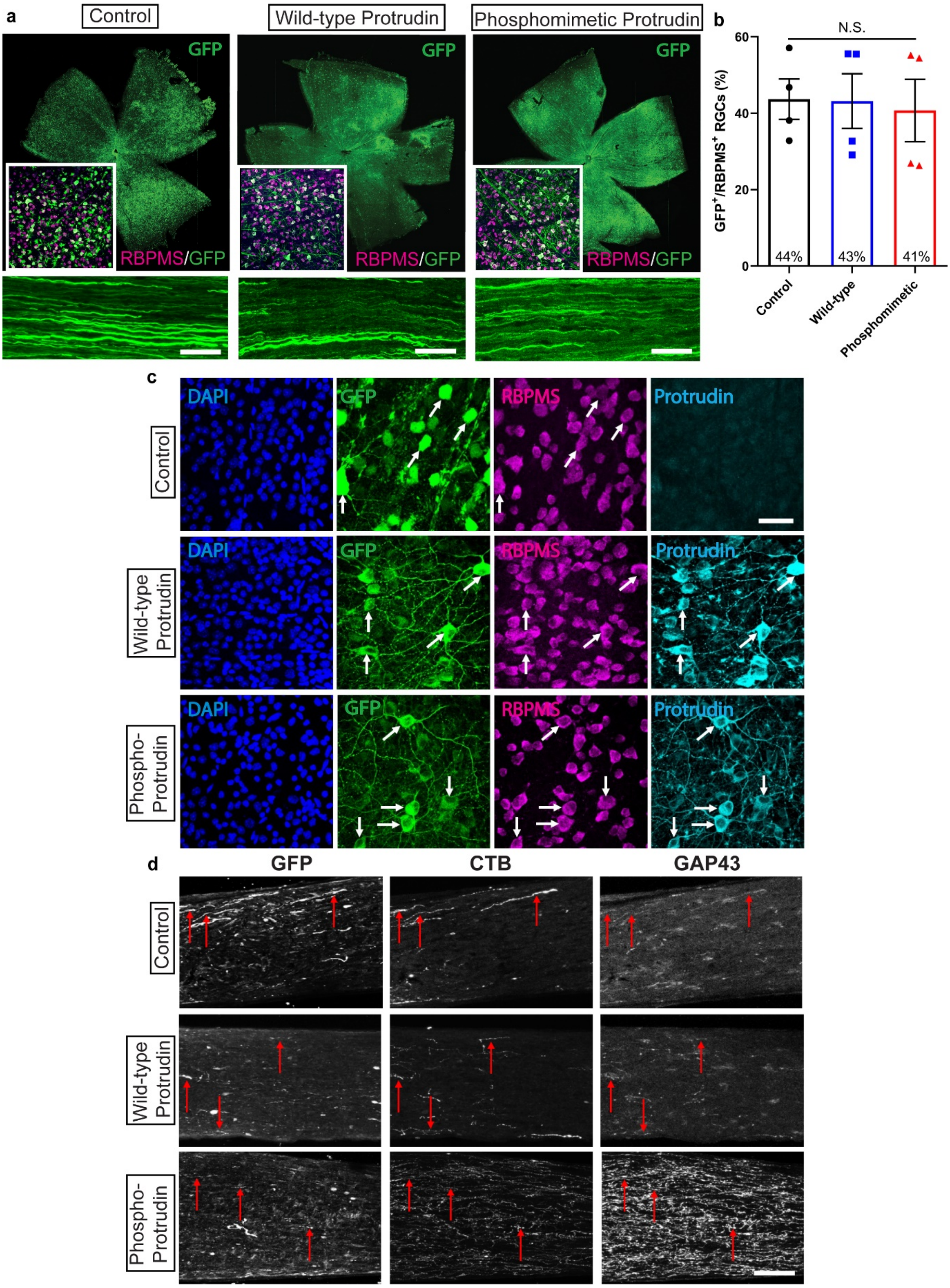

Supplementary Figure 7. Protrudin viruses are expressed in RGCs throughout the retina and the optic nerve and regenerating axons are GAP43 and CTB positive. (**a**) Retinal wholemounts or axonal sections showing GFP-positive cells which have been transduced with one of three

viruses: control GFP, wild-type Protrudin or phosphomimetic Protrudin-GFP. Retinas were immunostained for RBPMS - retinal ganglion cell marker (magenta) showing co-localization between the virally infected cells (GFP) and RGCs. Scale bar is 20 µm in axonal sections. (**b**) Robust viral expression in RGC-positive cells was detected for all constructs (n = 4 animals per condition) (*Fisher's exact* test with analysis of stack of p values and Bonferroni-Dunn multiple comparison test). Error bars represent mean ± SEM. (**c**) Retinal sections showing co-localization between RBPMS (magenta) and GFP (green) and elevated Protrudin levels (cyan) as detected by immunohistochemistry. Scale bars are 20 µm. (**d**) Immunolabelled optic nerve sections for GAP43, CTB and GFP. Red arrows show regenerating axons where GFP, CTB and GAP43 all co-localize. Scale bars are 10 µm.

Supplementary Tables

Oligonucleotides	
Name	Sequence
Phosphomimetic ZFYVE27 (S14D) – sense	TGGGCCGGAGCTGGACCCCAGCGTGATG
Phosphomimetic ZFYVE27 (S14D) – anti-sense	CATCACGCTGGGGTCCAGCTCCGGCCCA
Phosphomimetic ZFYVE27 (S25D) – sense	GGTAGGAAAAGGTGGATCCTCCAGGGGA GCCTCG
Phosphomimetic ZFYVE27 (S25D) – anti-sense	CGAGGCTCCCCTGGAGGATCCACCTTTTC CTACC
Phosphomimetic ZFYVE27 (S32D) – sense	TCCACCTTTTCCTACCAAGGACCCAGCGT TTGACCTTTTC
Phosphomimetic ZFYVE27 (S32D) – anti-sense	GAAAAGGTCAAACGCTGGGTCCTTGGTA GGAAAAGGTGGA
Phosphomimetic ZFYVE27 (T261D) –sense	GTGGGTGTGAGGGCAGGATCGCTGTCCA TCAGACCAT
Phosphomimetic ZFYVE27 (T261D) –sense	ATGGTCTGATGGACAGCGATCCTGCCCTC ACACCCAC
FYVE Domain Mutant - sense	CACCTTCTCAGTGCTGAAGGCGAGGGCG AGCTGCAGTAATTGTGGA
FYVE Domain Mutant – anti-sense	TCCACAATTACTGCAGCTCGCCCTCGCCT TCAGCACTGAGAAGGTG
FFAT Domain Mutant – sense	CAGATGAAGAGTTTAAAGCTGCGATTGA GGAGACCC
FFAT Domain Mutant – anti-sense	GGGTCTCCTCAATCGCAGCTTTAAACTCT TCATCTG
RBD Domain Mutant – sense	GTTCTCTCCTATAAACGTTTAGAAATATA TTTGCTCAGGTGGCAGA
RBD Domain Mutant – anti-sense	TCTGCCACCTGAGCAAATATATTTCTAAA CGTTTATAGGAGAGAAC
KIF5/VAPA Domain Mutant – sense	GAGAGTCTCTCTTCCCAGTTCTCAGTGCT GAAGAAG
KIF5/VAPA Domain Mutant – anti-sense	CTTCTTCAGCACTGAGAACTGGGAAGAG AGACTCTC
TM1-3 Domain Mutant 1	TGCTCAGGAATGAGGGTGCATGGTACTC AG
TM1-3 Domain Mutant 2	GCAGTTCATTCAGGGCACCGGACAGG
TM1-3 Domain Mutant 3	GCCCTGAATGAACTGCAAGACGAGGC
TM1-3 Domain Mutant 4	CCCTCATTCCTGAGCAAGTATCGAACAC
TM1-3 Domain Mutant 5	AGAACCCCGGGAATGTGGAGTTCTTCCG AG
TM1-3 Domain Mutant 6	GCAGTTCATTCAGGGCACCGGACAGG

TM1-3 Domain Mutant 7	GCCCTGAATGAACTGCAAGACGAGGC
TM1-3 Domain Mutant 8	ACCTCCTGATTCCTGAGCAAGTATCGAAC
TM1-3 Domain Mutant 9	CTCAGGAATCAGGAGGTTTGCCGGGCA
TM1-3 Domain Mutant 10	ACATTCCCGGGGTTCTCCCAGTGCAG

Supplementary Table 1. Table containing all primers used for cloning of Protrudin constructs.
