# Supplementary material for: Protrudin functions from the endoplasmic reticulum to support axon regeneration in the adult CNS
Source: Nat Commun. 2020 Nov 5;11:5614. doi: 10.1038/s41467-020-19436-y (PMC7645621; doi:10.1038/s41467-020-19436-y)
Supplement: Supplementary file 3 — Description of Additional Supplementary Files [file 41467_2020_19436_MOESM3_ESM.pdf]

Description of Additional Supplementary Files

Supplementary Movie 1. Example videos showing a non-regenerating axon expressing mCherry control plasmid and regenerating axons expressing either wild-type or phosphomimetic Protrudin after laser axotomy over a 14-hour period of imaging.
