# Supplementary material for: Protrudin functions from the endoplasmic reticulum to support axon regeneration in the adult CNS
Source: Nat Commun. 2020 Nov 5;11:5614. doi: 10.1038/s41467-020-19436-y (PMC7645621; doi:10.1038/s41467-020-19436-y)
Supplement: Supplementary file 5 — Reporting Summary [file 41467_2020_19436_MOESM5_ESM.pdf]

nature research

Corresponding author(s): Dr Veselina Petrova (vp351@cam.ac.uk)

Last updated by author(s): 21/09/2020

Reporting Summary

Nature Research wishes to improve the reproducibility of the work that we publish. This form provides structure for consistency and transparency in reporting. For further information on Nature Research policies, see Authors & Referees and the Editorial Policy Checklist .

Statistics

For all statistical analyses, confirm that the following items are present in the figure legend, table legend, main text, or Methods section.

n/a	Confirmed	
☐	☒	The exact sample size (*n*) for each experimental group/condition, given as a discrete number and unit of measurement
☐	☒	A statement on whether measurements were taken from distinct samples or whether the same sample was measured repeatedly
☐	☒	The statistical test(s) used AND whether they are one- or two-sided<br>*Only common tests should be described solely by name; describe more complex techniques in the Methods section.*
☐	☒	A description of all covariates tested
☐	☒	A description of any assumptions or corrections, such as tests of normality and adjustment for multiple comparisons
☐	☒	A full description of the statistical parameters including central tendency (e.g. means) or other basic estimates (e.g. regression coefficient) AND variation (e.g. standard deviation) or associated estimates of uncertainty (e.g. confidence intervals)
☐	☒	For null hypothesis testing, the test statistic (e.g. *F*, *t*, *r*) with confidence intervals, effect sizes, degrees of freedom and *P* value noted<br>*Give P values as exact values whenever suitable.*
☒	☐	For Bayesian analysis, information on the choice of priors and Markov chain Monte Carlo settings
☒	☐	For hierarchical and complex designs, identification of the appropriate level for tests and full reporting of outcomes
☒	☐	Estimates of effect sizes (e.g. Cohen's *d*, Pearson's *r*), indicating how they were calculated

Our web collection on statistics for biologists contains articles on many of the points above.

Software and code

Policy information about availability of computer code

Data collection	Images of immunostained cells were taken with a confocal microscope (Leica DMI 4000 B) using LAS-AF software (Leica Application Suite, Version 2.7.3.9723). For Protrudin localisation and intensity measurements as well as for ER localisation at the growth cone, a z-stack of images was obtained through each cell by taking an image at every 0.5 um thickness and an average intensity z-projection was created in Fiji ImageJ software where fluorescence intensity was measure against background (Schindelin et al., 2012). In vitro axotomy videos and data analysis was also performed using LAS-AF software. Images of optic nerves and retinas were obtained using the ZEN Digital Imaging Suite. Live-cell imaging was performed using spinning disk confocal microscopy, using an Olympus IX70 microscope with a Hamamatsu EM-CCD Image-EM camera and a PerkinElmer Ultra-VIEW scanner. Videos were taken using Meta-Morph software (Version 7.6.1.0). Alliance (Version 16.05) software was used for detection of Western blot signal. BCA assays were analysed using Gen5.1 software (Version 5.1).
Data analysis	Data analysis was performed using GraphPad Prism software (Version 8.0). Sholl analysis was performed in SPSS software (Version 26).

For manuscripts utilizing custom algorithms or software that are central to the research but not yet described in published literature, software must be made available to editors/reviewers. We strongly encourage code deposition in a community repository (e.g. GitHub). See the Nature Research guidelines for submitting code & software for further information.

Data

Policy information about availability of data

All manuscripts must include a data availability statement. This statement should provide the following information, where applicable:

- Accession codes, unique identifiers, or web links for publicly available datasets
- A list of figures that have associated raw data
- A description of any restrictions on data availability

The RNA-sequencing datasets from peripheral DRG neurons in development and after injury, from retinal ganglion cells during development or from cultured rat primary cortical neurons used for analysis in this study have previously been deposited in NCBI Gene Expression Omnibus (accession numbers: GSE66128, GSE90654

and GSE92856, respectively). The rest of the data generated to support the findings of this study are available from the corresponding author upon reasonable request.

Field-specific reporting

Please select the one below that is the best fit for your research. If you are not sure, read the appropriate sections before making your selection.

- [x] Life sciences
- [ ] Behavioural & social sciences
- [ ] Ecological, evolutionary & environmental sciences

For a reference copy of the document with all sections, see nature.com/documents/nr-reporting-summary-flat.pdf

Life sciences study design

All studies must disclose on these points even when the disclosure is negative.

Sample size	Sample sizes for animal studies were calculated using R software according to Chow et al., 2008 where alpha was set to 0.05 and the statistical power to 80%. Full R outputs are available upon request. Sample size calculations using data from previous experiments and pilot studies were used and power calculations which were performed for previously published studies in R (Chow et al., 2008). The cell culture data presented here used the sample sizes which had been calculated for these previously published studies.
Data exclusions	No data were excluded from the analyses.
Replication	Complete experiments were repeated a minimum of three times under controlled conditions and variation was tested between individual experiments. No significant variation was found between different series of the same experiments. Control and experimental groups were tested under identical conditions.
Randomization	Different viral treatments were assigned to animals randomly. Cell culture samples were assigned randomly, with control and experimental groups analysed in identical conditions to minimise potential covariates. For example, a typical laser axotomy experiment would run across four to five days (DIV13-17), with four experimental groups analysed. We took steps to ensure that no particular group was analysed on the same day. In this way samples would be analysed on a different day for each replicate of the experiments.
Blinding	The investigators were blinded during data collection and analyses.

Reporting for specific materials, systems and methods

We require information from authors about some types of materials, experimental systems and methods used in many studies. Here, indicate whether each material, system or method listed is relevant to your study. If you are not sure if a list item applies to your research, read the appropriate section before selecting a response.

Materials & experimental systems

n/a	Involved in the study	
[ ]	[x]	Antibodies
[ ]	[x]	Eukaryotic cell lines
[x]	[ ]	Palaeontology
[ ]	[x]	Animals and other organisms
[x]	[ ]	Human research participants
[x]	[ ]	Clinical data

Methods

n/a	Involved in the study	
[x]	[ ]	ChIP-seq
[x]	[ ]	Flow cytometry
[x]	[ ]	MRI-based neuroimaging

Antibodies

Antibodies used

1. Rabbit anti-Zfyve27 (ProteinTech, catalogue number: 12680-1-AP, RRID: AB_10640298)
2. Mouse Pan-Neurofascin (extracellular) (UC Davis/NIH NeuroMab Facility, catalogue number: 75-172, RRID: AB_2282826)
3. Rabbit anti-Reticulon 4 (Novus Biologicals, catalogue number: NB100-56681, RRID: AB_838641)
4. Rabbit anti-RBPMS (PhosphoSolutions, catalogue number: 1830-RBPMS, RRID: AB_2492225)
5. Mouse anti-mCherry (ClonTech, catalogue number: 632543, RRID: AB_2307319)
6. DAPI (ThermoFisher Scientific, catalogue number: D3751; RRID: AB_2307445)
7. Sheep anti-GAP43 (kind donation from the Benowitz lab, PMID: 3339416, Benowitz et al., 1988)
8. Mouse monoclonal anti-beta actin (C4) HRP conjugated (Santa Cruz, sc-47778, RRID: AB_2714189)

Goat anti-rabbit IgG HRP conjugated (Sigma, A4914, RRID: AB_258207)
Alexa Fluor 647 Phalloidin (ThermoFisher Scientific, catalogue number: A22287, RRID: AB_2620155)
Goat anti-rabbit 488 (ThermoFisher Scientific, catalogue number: A27034; RRID: AB_2536097)
Goat anti-rabbit 568 (ThermoFisher Scientific, catalogue number: A-11011; RRID: AB_143157)
Goat anti-rabbit 647 (ThermoFisher Scientific, catalogue number: A27040; RRID: AB_2536101)
Goat anti-mouse 647 (ThermoFisher Scientific, catalogue number A-21235; RRID: AB_2535804)

	Goat anti-mouse 568 (ThermoFisher Scientific, catalogue number: A-11031; RRID: AB_144696)
Validation	Antibody 1 was validated in primary rat cortical neurons in the current study using human Zfyve27 overexpression as a positive control (previously published data on antibody provider website) and no secondary antibody for immunofluorescence (Fig. SI and data not shown). Antibody 1 was previously validated in Western blots as described on the provider's website and in previously published literature (Zhang et al., 2017; Chang et al., 2013). Antibodies 2, 3 and 5 have previously been validated in rat primary neurons for immunocytochemistry according to their manufacturers' websites. Antibody 4 has been previously validated in mouse for immunohistochemistry as describe on the manufacturer's websites. Antibody 7 has previously been validated in Benowitz et al., Journal of Neuroscience, 1988 for immunohistochemistry in adult rat brain and in Pearson et al., eLife, 2018 for immunohistochemistry in mouse optic nerve. Antibody 6 was validated for immunohistochemistry and immunocytochemistry in rat and mouse according to the manufacturer's website and has previously been validated for these techniques in Pearson et al., eLife, 2018 and in Lawler et al., Muscle and nerve, 2006. Antibody 8 was recommended and validated for Western blot use as per the manufacturer website.

Eukaryotic cell lines

Policy information about cell lines

Cell line source(s)	PC12 cells and HeLa cells were both acquired from the European Collection of Cell Cultures (ECACC).
Authentication	PC12 cells were validated by testing differentiation to a neuronal phenotype after stimulation with NGF using an established protocol (Eva et al., J. Neurosci., 2010). HeLa cells were previously validated by STR profiling (Eurofins Genomics) as described in Menzies et al., eLife, 2018.
Mycoplasma contamination	Cells were confirmed mycoplasma free by PCR, using the SigmaAldrich LookOut mycoplasma PCR detection kit.
Commonly misidentified lines (See ICLAC register)	No commonly misidentified cell lines were used in this study.

Animals and other organisms

Policy information about studies involving animals; ARRIVE guidelines recommended for reporting animal research

Laboratory animals	Female C57Bl/6 mice aged 6-8 weeks (Charles River) were housed in a pathogen-free facility with free access to food and a standard 12 h light/dark cycle. Female pregnant Sprague Dawley rats (8-12 weeks old) were obtained from Charles River Laboratories, and embryos (both genders) at E18 stage of development were used for primary cultures of cortical neurons. Animals were housed at ambient temperature of 18-23 degrees C and 40-60% humidity with food access ad libitum.
Wild animals	The study did not involve wild animals.
Field-collected samples	The study did not involve samples collected from the field.
Ethics oversight	All procedures were performed in accordance with protocols approved by the Institutional Animal Care and Use Committee (IACUC) at the National Institutes of Health, by the UK Home Office regulations for the care and use of laboratory animals under the UK Animals (Scientific Procedures) Act (1986) and in accordance with the Swedish Board of Agriculture guidelines and were approved by the Karolinska Institutet Animal Care Committee.

Note that full information on the approval of the study protocol must also be provided in the manuscript.
